# Prognostic impact of tumor infiltrating CD8+ T cells in association with cell proliferation in ovarian cancer patients - a study of the OVCAD consortium

**DOI:** 10.1186/1471-2407-13-422

**Published:** 2013-09-17

**Authors:** Anna Bachmayr-Heyda, Stefanie Aust, Georg Heinze, Stephan Polterauer, Christoph Grimm, Elena Ioana Braicu, Jalid Sehouli, Sandrina Lambrechts, Ignace Vergote, Sven Mahner, Dietmar Pils, Eva Schuster, Theresia Thalhammer, Reinhard Horvat, Carsten Denkert, Robert Zeillinger, Dan Cacsire Castillo-Tong

**Affiliations:** 1 Department of Obstetrics and Gynecology, Molecular Oncology Group, Comprehensive Cancer Center, Medical University of Vienna, Waehringer Guertel 18-20, A-1090 Vienna, Austria; 2 Section for Clinical Biometrics, Center for Medical Statistics, Informatics and Intelligent Systems, Medical University of Vienna, Spitalgasse 23, A-1090 Vienna, Austria; 3 Department of Gynecology, European Competence Center for Ovarian Cancer, Campus Virchow Klinikum, Charité - Universitätsmedizin Berlin, Augustenburger Platz 1, D-13353 Berlin, Germany; 4 Division of Gynaecological Oncology, Department of Obstetrics and Gynaecology, Universitaire Ziekenhuizen Leuven, Katholieke Universiteit Leuven, UZ Leuven, Herestraat 49, B-3000 Leuven, Belgium; 5 Department of Gynecology and Gynecologic Oncology, University Medical Center Hamburg-Eppendorf, Martinistraße 52, D-20246 Hamburg, Germany; 6 Department of Pathophysiology, Center for Pathophysiology and Allergy Research, Medical University of Vienna, Waehringer Guertel 18-20, A-1090 Vienna, Austria; 7 Clinical Institute of Pathology, Medical University of Vienna, Waehringer Guertel 18-20, A-1090 Vienna, Austria; 8 Institute of Pathology, Charité University Hospital, Charitéplatz 1, D-10117 Berlin, Germany; 9 Ludwig Boltzmann Cluster Translational Oncology, General Hospital of Vienna, Waehringer Guertel 18-20, A-1090 Vienna, Austria

**Keywords:** Epithelial ovarian cancer, Cytotoxic T cells, Tumor proliferation, Prognostic impact, Residual tumor

## Abstract

**Background:**

Epithelial ovarian cancer is one of the most lethal gynecologic malignancies. Clinicopathological factors do not permit precise prognosis and cannot provide guidance to specific treatments. In this study we assessed tumor infiltrating CD8+ T cells in association with Ki67 proliferation index and evaluated their prognostic impact in EOC samples.

**Methods:**

CD8+ cells and Ki67 proliferation index were immunohistochemically determined on tissue microarrays including 203 primary epithelial ovarian tumors. Additionally, CD8 gene expression was assessed with RT-qPCR. Correlations were analyzed using Pearson’s correlation coefficients, ANOVA or T-test, or Fischer’s exact tests. Prognostic impact was evaluated using the Kaplan-Meier method and Cox regression model.

**Results:**

The density of CD8+ infiltrating lymphocytes did not correlate with tumor cell proliferation. Epithelial ovarian cancer patients with no Ki67+ cells in the tumor had a more than three times higher risk to die compared to the population with Ki67+ cells in the tumor (Hazard ratio (HR) = 3.34, 95%CI 1.59-7.04). High CD8+ cell infiltration was associated with improved overall survival (HR = 0.82, 95%CI 0.73-0.92).

**Conclusions:**

The density of tumor infiltrating lymphocytes is independent of tumor cell proliferation. Ovarian cancer patients with Ki67- tumors showed a significantly reduced overall survival, presumably due to no or poor response to platinum-based chemotherapy. Moreover, the association of high densities of tumor infiltrating cytotoxic T lymphocytes with a better overall survival was confirmed.

## Background

Epithelial ovarian cancer (EOC) is one of the most lethal gynecologic malignancies with 67,000 new cases and 42,000 deaths in Europe per year [1]. Despite increasing knowledge in the etiology of ovarian cancer and the improvements in surgery and chemotherapy, there has been little change in the survival of patients. Clinicopathological factors do not permit precise prognosis for the disease and thus cannot provide guidance to specific treatments.

Proliferation is one of the most important hallmarks of cancer and has been reported to have impact on prognosis in various malignancies. High cell proliferation, determined mostly by biomarkers such as Ki67, has been correlated with occurrence of metastases and subsequent worse clinical outcome for melanoma patients [2]. In contrary, in colorectal and gastric cancer, Ki67 has also been associated with improved overall survival and relapse-free survival [3,4]. In ovarian cancer, Ki67 proliferation index has been associated with advanced stage, high grade and complete responsiveness to first-line chemotherapy. Ki67 has also been reported as independent prognostic factor for poor overall and progression-free survival [5-7].

Infiltrating lymphocytes are frequently found in tumor tissues, indicating an ongoing host immune response. The prognostic value of tumor infiltrating lymphocytes on the clinical outcome has been assessed in a variety of cancer entities [8-10]. Various studies have reported a survival advantage associated with the presence of tumor infiltrating T cells (CD3) and cytotoxic T cells (CD8) [11]. However, other studies revealed a non-significant prognostic value of CD3+ and/or CD8+ T lymphocytes [10,12,13]. In EOC, tumor infiltrating CD8+ cells have been described to play a major role in antitumoral activity and survival [14-17].

We hypothesize that the outcome of cancer patients is a result of interactions of tumor cell proliferation and host immune reaction. The proliferative potential of tumors may influence leukocyte infiltration. In breast cancer, CD8+ T cells were found to be less abundant in the tumor microenvironment of highly proliferating tumors [18]. Another study confirmed the prognostic impact of infiltrating lymphocytes only in rapidly proliferating breast cancer tissues [19]. For EOC, the association of cancer cell proliferation and host immune response has seldom been addressed.

In this study, we assessed tumor infiltrating CD8+ T cells as one of the important factors in the adaptive immune system in association with Ki67 expression that reflects the tumor proliferation by immunohistochemistry (IHC) and RT-qPCR and evaluated their prognostic impact in EOC.

## Methods

### Patient information

203 patients with epithelial FIGO stage II to IV ovarian cancer from OVCAD (FP6 EU-project, Ovarian Cancer: Diagnosis of a silent killer, no. 018698, http://www.ovcad.eu) were included in the study. Patients have been recruited from 2005 to 2008 in the Department of Gynecology at Charité, Campus Virchow-Klinikum, Medical University Berlin, Germany (64); Department of Obstetrics and Gynecology and Gynecologic Oncology, University Hospital Leuven, Belgium (54); Department of Gynecology, University Medical Center Hamburg-Eppendorf, Germany (38); Department of Obstetrics and Gynecology, Medical University of Vienna, Austria (37); Department of Gynecology and Obstetrics, Innsbruck Medical University, Austria (10). Informed consents were obtained from all patients. All processes were approved by the respective local ethical committee (EK207/2003, ML2524, HEK190504, EK366, EK260). Patients were treated with cytoreductive surgery and platinum-based chemotherapy. Tumor tissues were obtained during primary surgery and before any chemotherapeutic treatment. Residual tumor load was defined as negative if macroscopically absent. Overall survival (OS) was defined as the time from diagnosis to death from any disease-related cause. The survival times of patients alive at their last follow-up visit were treated as censored. Progression-free survival (PFS) was defined as the time from diagnosis until progression of disease, censoring patients who were recurrence-free at their last follow-up visit, and excluding patients with refractory disease, defined as progression of disease while receiving first line platinum-based therapy or within four weeks of the last platinum application [20]. Progression was defined by radiological diagnosis according to the RECIST criteria or as doubling of the nadir serum CA-125 [21]. Experienced gynecological oncologists and pathologists performed the clinical and histopathological evaluations.

### Tissue microarray

Tissue microarrays (TMA) were assembled using two one mm^2^ tissue cores of the same primary tumor tissue block per patient. For each tissue sample, representative tumor areas were marked on the hematoxylin-eosin-stained section. The cores were punched from different selected areas of each sample using a tissue micro-arrayer (Beecher Instruments, Woodland, CA, USA) and embedded in a new paraffin block. Regarding tumour infiltrating CD8+ cells, the mean value of the two TMA cores were confirmed to be representative of the whole tumour tissue [22].

### Immunohistochemistry

Antigen heat retrieval was performed by microwaving the slides for 15 minutes in EDTA (1 mM, pH 8.0) and Dako Target Retrieval Solution (Dako, Denmark) for the CD8 and Ki67 staining, respectively. Slides were then cooled to room temperature and quenched for endogenous peroxidase. Blocking solution (Ultra V Block; TA-015HP, Thermo Fisher Scientific, USA) was applied prior to incubation with monoclonal mouse antibodies against CD8 (1:1000; clone C8/144B, code M 7103, Dako, Denmark) and Ki67 (1:75, clone MIB-1, code M7240, Dako, Denmark) overnight at 4°C. UltraVision detection system (Thermo Fisher Scientific, USA) and the Dako LSAB System (Dako, Denmark) were used for CD8 and Ki67, respectively, according to the manufacturers’ instructions. The CD8 stained sections were incubated with Primary Antibody Enhancer (TL-015-PB, Thermo Fisher Scientific, USA), followed by horseradish peroxidase (HRP) Polymer (HRP Polymer; TL-015-PH, Thermo Fisher Scientific, USA); for Ki67 staining, Dako Biotinylated Link (K0675, Dako, Denmark), followed by Dako Streptavidin-HRP (K0675, Dako, Denmark) was applied. The slides were stained with diamino-benzidine (DAB, 1:50 in DAB Substrate Buffer, K0673, Dako, Denmark) and counterstained with hematoxylin. Lymph node and normal colon tissue specimens were used as positive controls for CD8 and Ki67, respectively. Mouse IgG1 (1:75; Negative Control Mouse IgG1, code X0931, Dako, Denmark) was used as isotype control.

Slides were digitally photographed with the TissueFAXS system (version 2.0.4.0147, TissueGnostics, Austria) using an x20 objective lens. HistoQuest software (version 3.0.3.0161, TissueGnostics, Austria) was used for the detection and quantification of CD8+ cells. The TissueFAXS detection and quantification method was recently applied in other human cancer entities [23] and is based on techniques described and validated by Steiner et al. [24]. The CD8+ cell density (cells/mm^2^) was determined only in epithelial tumor tissue. To avoid false positive cell counting, a specific gate according to cell size and intensity of CD8 staining was set within which the cells were considered positive. These cells were randomly controlled by applying a function permitting the visualization of the corresponding cells on the digital picture.

Scoring of the Ki67 proliferation index was evaluated manually by two independent observers determining the percentage of Ki67+ cells in total epithelial tumor tissue.

### RNA extraction, cDNA synthesis and qPCR

Tumor tissues from 158 out of the 203 patients were available. About 30 mg fresh frozen tumor tissue was homogenized using a Mikro-Dismembrator U (B. Braun, Biotech International, Germany) and lysed in one ml Nucleic Acid Purification Lysis Solution (Applied Biosystems, Life Technologies, USA). Total RNA from the lysates was isolated with the ABI PRISM 6100 Nucleic Acid PrepStation (Tissue RNA isolation, Applied Biosystems, Life Technologies, USA) and quantified spectrophotometrically. The quality of RNA was assessed with an Agilent 2100 Bioanalyzer. RNA with an RNA Integrity Number >5 was used.

The cDNA synthesis was performed with the Omniscript Reverse Transcription Kit (QIAGEN, Netherlands) with 500 ng RNA according to manufacturer’s instructions. cDNA was diluted 1:2 with TE buffer and deposited at −80°C for further analysis. The qPCR was performed with the CD8A TaqMan Gene Expression Assay (Hs00233520_m1, Applied Biosystems, Life Technologies, USA) according to the manufacturer’s instructions. As a reference, gene expression of house-keeping gene GAPDH (Hs99999905_m1, Applied Biosystems, Life Technologies, USA) was measured. 2 μl cDNA, 0.4 μl TaqMan Gene Expression Assay, 4 μl 2x TaqMan Gene Expression MasterMix (Applied Biosystems, Life Technologies, USA) and 1.6 μl H_2_O were used. The reaction mixture was pre-incubated at 50°C for two minutes and at 95°C for ten minutes, followed by 40 cycles of two step incubation at 95°C for 15 seconds and at 60°C for one minute. Each PCR was performed in duplicates.

### Statistical analyses

Raw CD8+ cell density values were log_2_-transformed to achieve normal distribution. For each patient, the mean value of the two cores was calculated. To evaluate the RT-qPCR data, the mean value of the duplicate expression values (Ct values) was calculated and normalized with the mean Ct value of the reference gene GAPDH. Differences between plates were corrected with a calibrator. Finally the normalized values were multiplied by −1 to be interpretable as log_2_-expression (relative expression values). Statistical analyses were performed with SPSS (version 19, Chicago, USA) and SAS (version 9.3, 2011 SAS Institute Inc., Cary, NC, USA). Correlation of continuous variables (CD8+ cell density and relative expression values, Ki67 proliferation index and age) was assessed by Pearson’s correlation coefficients. Continuous variables were compared between groups by ANOVA or T-test. The association of categorical variables was evaluated by Fisher’s exact tests. Cumulative survival probabilities were calculated by the Kaplan-Meier method. Univariate and multivariable Cox proportional hazards regression analysis was used to evaluate the marginal and adjusted association of CD8+ cell density, percentage of Ki67 proliferation index, CD8 relative expression values and the clinicopathological factors age, FIGO stage and residual tumor with survival [25]. For multivariable regression, the multivariable fractional polynomial approach was used, which evaluates possible non-linear effects of continuous variables such as CD8+ cell density or Ki67 by a set of parsimonious transformations [26]. Because of the relatively low number of patients with FIGO II (9), we modeled FIGO stage as ordinal rather than categorical variable, assuming the same hazard ratio between FIGO IV and III as between FIGO III and II. This strategy provides more stable results than with categorical modeling of FIGO stage. Pairwise interactions between CD8+ cell density and other variables were tested by assessing significance of corresponding product terms. Two-sided p values <0.05 were considered statistically significant in all the analyses.

## Results

### Clinical outcome of the patients

Clinical and pathological characteristics of tumors of the 203 EOC patients are depicted in Table 1. The median age at time of diagnosis was 56 years (range 18–85 years). Median follow-up time was 48 months (25^th^ percentile, 39 months; 75^th^ percentile, 57 months). 12 patients (6%) with refractory disease were excluded from PFS analyses. 95 patients (47%) died within the observation period and 139 (77%) patients had a recurrence. Median PFS was 19 months (25^th^ percentile, 11 months; 75^th^ percentile, 36 months) and median OS was 50 months (25^th^ percentile, 25 months; 75^th^ percentile, not reached).

**Table 1:** Clinicopathological characteristics of the tumors

** Characteristics**	**N (%)**
**Histology**	
Serous	179 (88.2)
Non-serous^1^	24 (11.8)
**FIGO stage**	
II	9 (4.4)
III	160 (78.8)
IV	34 (16.7)
**Grade**	
1	7 (3.4)
2	45 (22.2)
3	150 (73.9)
Unkown	1 (0.5)
**Residual tumor**	
No	141 (69.5)
Yes	62 (30.5)
**Total**	203 (100.0)

^1^8 endometrioid, 9 mixed epithelial, 1 mucinous, 4 undifferentiated, 2 clear cell.

### No correlation between tumor CD8+ infiltrating lymphocytes and Ki67 proliferation index

**Figure 1:**
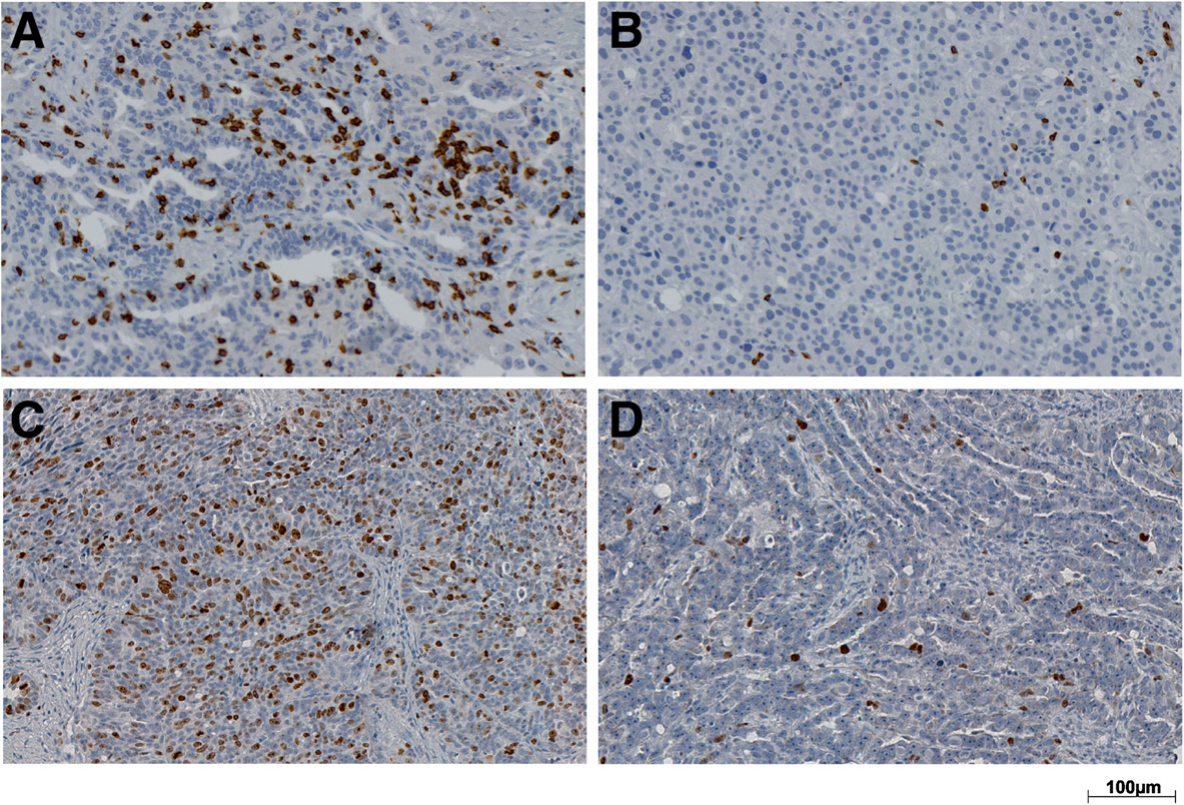
**Representative CD8 and Ki67 immunohistochemical staining in epithelial ovarian cancer. A** and **B**: high and low CD8+ cell infiltration, respectively; **C** and **D**: high and low Ki67 proliferation index, respectively; optical magnification x200.

Representative immunohistochemical staining of CD8 and Ki67 is shown in Figure 1. Intraepithelial CD8+ cells were present in all samples with values ranging from 3 to 2,257 cells/mm^2^ and a median of 137 cells/mm^2^. In 4% of tumors, less than ten CD8+ cells/mm^2^ were observed. Log_2_-transformed CD8+ cell densities showed normal distribution. Ki67 proliferation indices ranged from 0% to 90% with a median of 30%. In 5% of tumors, no Ki67+ cells were detected. The density of CD8+ infiltrating lymphocytes did not correlate with Ki67 proliferation index (data not shown).

### Better overall survival of ovarian cancer patients with Ki67+ tumors and high density of tumor infiltrating CD8+ lymphocytes

**Figure 2:**
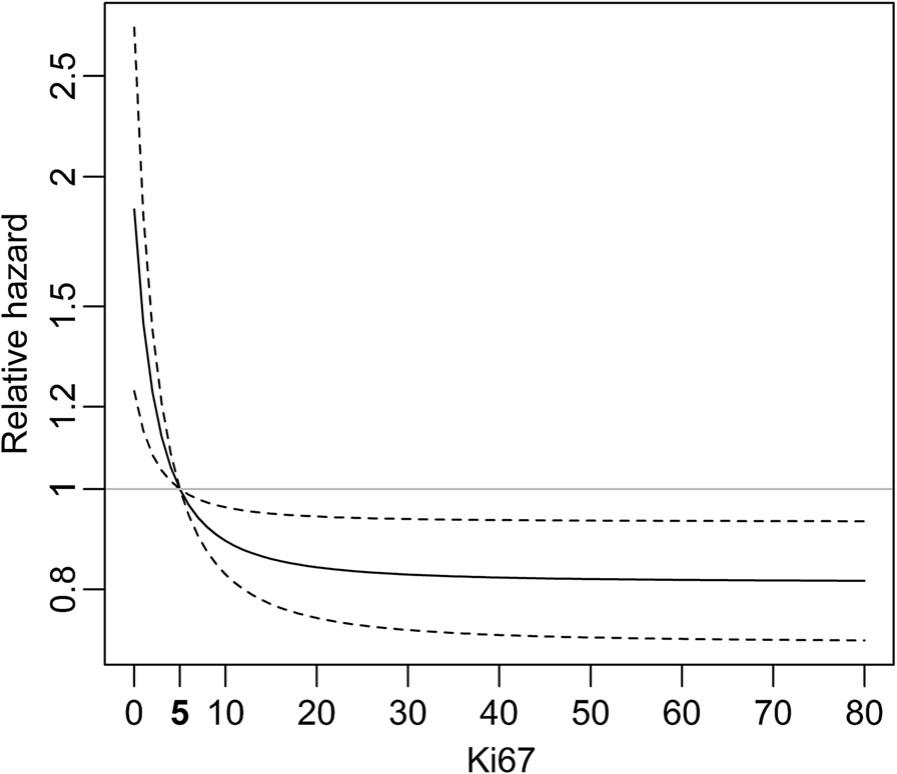
**Non-linear association of Ki67 proliferation index with overall survival.** Association adjusted for age, FIGO, residual tumor and CD8+ cell infiltration; relative hazard compared to a reference level of Ki67 = 5%; X-axis indicates Ki67 proliferation index.

Fractional polynomial modeling of the continuous factors age, Ki67 proliferation index and the density of CD8+ cells confirmed linearity for age and CD8, whereas a non-linear association of Ki67 with survival was revealed. Visualizing the shape of this non-linearity revealed that the mortality risk was sharply increased for patients with 0% Ki67+ cells, while it was constantly low for patients with more than 5% Ki67+ cells (Figure 2). Therefore, it is reasonable to dichotomize Ki67 at a cutoff of 5% resulting in ten patients with 0% Ki67+ cells (Ki67- tumors) and 190 patients with ≥5% Ki67+ cells (Ki67+ tumors). This dichotomization was used for OS as well as PFS analyses.

Multivariable Cox proportional-hazards regression analyses revealed age, FIGO stage, residual tumor, CD8+ cells and Ki67 proliferation index to be significantly and independently associated with OS. The mortality risk increased by about 26% per each ten years of age (hazard ratio (HR) = 1.26, 95% CI: 1.07-1.48) and was more than doubled if a patient had a higher FIGO stage (IV vs. III, or III vs. II: HR = 2.18, 95% CI 1.38-3.45). Patients with residual tumor after cytoreductive surgery had an almost 90% higher risk to die than optimally debulked patients (HR = 1.87, 95%CI 1.21-2.88). For CD8+ cell density, the mortality risk decreased by approximately 18% with each doubling of cells (HR = 0.82, 95%CI 0.73-0.92). Patients with Ki67- tumors (0% Ki67+ cells) had a significantly poorer OS than those with Ki67+ tumors (≥5% Ki67+ cells) (HR 3.34, 95%CI 1.59-7.04, Table 2A).

**Table 2:** **Results of cox regression analyses**^**1**^

**A) Prognostic values of various parameters on overall survival**				
**N = 197 (93 events)**	**Univariate**		**Multivariable**	
	**HR (CI95%)**	**p**	**HR (CI95%)**	**p**
Age (continuous, per decade)	**1.35** (1.14-1.60)	**<0.001**	**1.26** (1.07-1.48)	**0.006**
FIGO (ordinal, per stage)	**2.22** (1.44-3.42)	**<0.001**	**2.18** (1.38-3.45)	**<0.001**
Residual tumor (yes vs. no)	**2.01** (1.32-3.06)	**0.001**	**1.87** (1.21-2.88)	**0.005**
CD8+ cell infiltration (continuous, per doubling)	**0.86** (0.77-0.96)	**0.006**	**0.82** (0.73-0.92)	**<0.001**
Ki67+ tumor cells (<5% vs. ≥5%)	**3.27** (1.57-6.83)	**0.002**	**3.34** (1.59-7.04)	**0.001**
**B) Prognostic values of various parameters on progression-free survival**				
**N = 180 (139 events)**	**Univariate**		**Multivariable**	
	**HR (CI95%)**	**p**	**HR (CI95%)**	**p**
Age (continuous, per decade)	**1.18** (1.03-1.34)	**0.016**	1.08 (0.94-1.23)	0.268
FIGO (ordinal, per stage)	**2.51** (1.72-3.67)	**<0.001**	**2.19** (1.43-3.35)	**<0.001**
Residual tumor (yes vs. no)	**2.07** (1.44-2.98)	**<0.001**	**1.64** (1.11-2.43)	**0.013**
CD8 cell infiltration (continuous, per doubling)	0.97 (0.89-1.05)	0.447	0.92 (0.84-1.01)	0.074
Ki67 (<5% vs. ≥5%)	1.63 (0.76-3.49)	0.209	1.51 (0.69-3.28)	0.299

^1^HR and p values of statistically significant parameters presented in bold.

For PFS, FIGO stage (HR 2.19, 95%CI 1.43-3.35) and residual tumor (HR 1.64, 95%CI 1.11-2.43) were confirmed as independent prognostic factors. Ki67 proliferation index and tumor infiltrating CD8+ lymphocytes had no significant impact on PFS (Table 2B).

**Figure 3:**
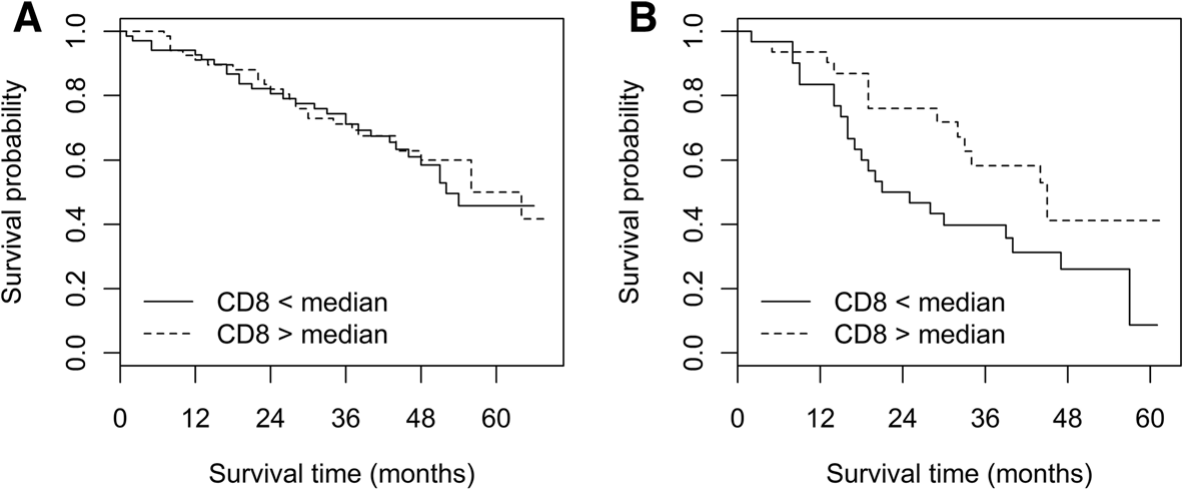
**Kaplan-Meier curves showing the association between CD8+ cell infiltration and overall survival of patients.** CD8+ cell infiltration (low versus high, cut-off value at median); **A**: in optimally debulked patients; log-rank test, p = 0.596; **B**: in patients with residual tumor; log-rank test, p = 0.055.

Since infiltrating CD8+ lymphocytes are removed together with the tumor mass, we assumed that their effect might be less important in optimally debulked patients (n = 141) compared to patients with residual tumor (n = 62). Similarly, different benefits from surgical cytoreduction were observed regarding CD8+ cell tumor infiltration [27]. Indeed, the Kaplan-Meier curves with dichotomized CD8+ cell infiltration values (cutoff median) show that in non-optimally debulked patients, low CD8+ cell infiltration was associated with worse OS compared to those with high CD8+ cell infiltration (p = 0.055, Figure 3B). In contrast, in women without residual tumor, CD8+ cell infiltration did not influence OS (Figure 3A). Accordingly, univariate Cox hazard regression analysis revealed a significant survival advantage of patients with high CD8+ cell tumor infiltration (cutoff median) in patients with residual tumor (high vs. low: HR = 0.46, 95% CI 0.24-0.88, p = 0.020), whereas CD8+ cell infiltration was not of prognostic value in patients without residual tumor (high vs. low: HR = 0.96, 95% CI 0.57-1.61, p = 0.864). A formal interaction test for CD8+ cell density and residual tumor did just not reach significance (p = 0.052).

### mRNA expression of CD8 was not correlated with the density of infiltrating CD8+ cells and had no prognostic impact

Both mRNA expression data and the immunohistochemistry results could be obtained from 158 patients. There was no correlation between the CD8 gene expression values and the CD8+ cell density values (R = 0.30, p < 0.001). Gene expression of CD8 had neither impact on OS nor on PFS (data not shown).

### Correlation of CD8 and Ki67 with clinicopathological factors

No associations of Ki67 proliferation index (either as continuous parameter or dichotomized at 5%), CD8+ cell density, or CD8 gene expression values with the clinicopathological parameters patients’ age, histological subtype, grade, stage, and residual tumor were found (data not shown). There was a significant correlation between FIGO stage and residual tumor (p = 0.012). No associations were found between the other clinicopathological factors (data not shown).

## Discussion

In this study, we found that a small population of ovarian cancer patients with no Ki67+ cells in the primary tumor had a more than three times higher risk to die compared to patients with ≥5% Ki67+ cells. Additionally, an association of CD8+ cell infiltration with improved OS was observed. Interestingly, these two tumor characteristics were not associated with each other.

To investigate the interaction of tumor cell proliferation and immunological components in the tumor microenvironment, we analyzed the correlation of Ki67 expression and tumor infiltrating CD8+ cells. No association was found, demonstrating that the proliferative ability of tumor cells is not essential for the infiltration of cytotoxic lymphocytes in human ovarian cancer tissue. This is in contrast to a previous study reporting a weak association between Ki67 expression and intraepithelial CD8+ cells [27]. This discrepancy could be derived from dichotomizing parameters at different cutoffs. In order to define the molecular characteristics of the tumor influencing the infiltration with leukocytes, various molecules implied in antigen processing and presentation, costimulation or leukocyte recruitment should be investigated.

An association of the proliferative status of ovarian tumors with survival has been reported before, using p21-activted kinase 4 (Pak4) [28] or cell cycle-related kinase (CCRK) as markers [29]. There are few reports about the prognostic value of the cell proliferation marker Ki67 in EOC. Rapidly proliferating tumors are expected to cause poor PFS in patients with residual tumor after cytoreductive surgery. We did not find such correlation, suggesting that tumor growth might be controlled by other mechanisms. Patients with Ki67- tumors had a strongly reduced overall survival, indirectly indicating that these tumors may have poor or no response to the chemotherapeutic drug. All patients included in this study received platinum-based chemotherapy, in which the platinum compounds cause crosslinking of DNA and trigger apoptosis of the tumor cells. Platinum-compounds, like other cytotoxic drugs, are believed to gain their specificity by preferentially killing highly proliferative cells. If residual tumor cells divide quickly, they will be killed by platinum and/or paclitaxel compounds when they enter mitosis. In contrast, slowly growing cells might survive chemotherapy. Consistently, correlations between clinical complete remission to first-line chemotherapy and high Ki67 proliferation index have been reported by other groups [7,30].

If women with Ki67- tumors were identified at the time of primary surgery, they could be selected for treatment with alternative drugs, such as angiogenesis inhibitors that reduce tumor growth by inhibiting blood vessel formation rather than targeting rapidly proliferating cells. Studies comprising larger cohorts of patients with low Ki67 proliferation indices are needed to validate the results described in this study.

The importance of tumor infiltrating lymphocytes in EOC has recently been investigated [8,15]. In accordance with previous results, we found a correlation of high CD8+ cell infiltration with improved OS [14-16]. The lack of prognostic impact on PFS agrees with most studies of tumor infiltrating lymphocytes in EOC [11]. Comparing the impact of CD8+ cell density between optimally and non-optimally debulked patients, only for the latter group an association trend between high CD8+ cell infiltration and improved OS was observed, but not for the former group. In accordance with this rationale, Adams et al. reported a more likely benefit from surgical debulking for patients with aggressive tumors having low lymphocyte infiltration and high Ki67 expression [27]. The formal interaction test between the factors residual tumor and CD8+ cell infiltration showed non-significance. So, our results should be regarded as hypothesis generating rather than hypothesis confirming and need to be validated in an independent patient cohort.

mRNA expression as prognostic markers has been studied in various tumors. For some genes little or no correlation between protein and mRNA expression was found [31,32]. In our study, the CD8 gene expression values obtained from RT-qPCR showed no correlation with the CD8 data generated by IHC. In addition, the prognostic impact of CD8+ cells could not be represented by CD8 gene expression. The differences may be attributed to the fact that mRNA was obtained from heterogeneous tumor tissue samples comprising not only epithelial tumor tissue, but also stroma and blood vessels, whereas in IHC only tumor infiltrating CD8+ cells in epithelial tumor areas were analyzed. These results indicate that gene expression measurement may not be suitable for infiltrating immune cells, at least if tumor tissues are not micro-dissected.

## Conclusions

In summary, we observed a significantly reduced survival of epithelial ovarian cancer patients with Ki67- tumors indicating a poor response to the chemotherapeutic drug. This small group of patients could benefit from treatment with alternative drugs. Moreover, a higher number of tumor infiltrating cytotoxic T lymphocytes was associated with a better overall survival, presumably due to the stronger effect in non-optimally debulked patients.

## Abbreviations

FIGO: International federation of gynecology and obstetrics; IHC: Immunohistochemistry; EOC: Epithelial ovarian cancer; TMA: Tissue microarrays; OS: Overall survival; PFS: Progression-free survival; HRP: Horseradish peroxidase; DAB: Diamino-benzidine; HR: Hazard ratio; Pak4: p21-activted kinase 4; CCRK: Cell cycle-related kinase.

## Competing interests

The authors declare that they have no competing interests.

## Authors’ contributions

AB carried out the immunohistochemical staining and drafted the manuscript. SA participated in the immunohistochemical staining and evaluation and in manuscript writing. GH performed the statistical analyses. SP, CG, EIB, JS, SL, IV, and SM contributed to study design, collected patients’ materials, clinical and patients’ information. DP participated in data interpretation and statistical analyses. ES performed the RNA purification and RT-PCR analysis. TT participated in the computerized detection and quantification of immunohistochemical stainings. RH performed the pathological examination and evaluated immunohistochemical stainings. CD performed the pathological examination and assembled the TMA. RZ participated in the design and coordination of the study. DC designed and coordinated the study and helped to draft the manuscript. All authors read and approved the final manuscript.

## Pre-publication history

The pre-publication history for this paper can be accessed here:

http://www.biomedcentral.com/1471-2407/13/422/prepub
